# Patient Self-Inflicted Lung Injury in ARDS: From Physiological Concept to Clinical Syndrome

**DOI:** 10.3390/jcm15041412

**Published:** 2026-02-11

**Authors:** Raffaele Merola, Patricia R. M. Rocco, Denise Battaglini

**Affiliations:** 1 Anesthesia and Intensive Care Medicine, Department of Critical Care, AORN Ospedali Dei Colli, 80131 Naples, Italy; 2 Laboratory of Pulmonary Investigation, Carlos Chagas Filho Institute of Biophysics, Federal University of Rio de Janeiro, Avenida Carlos Chagas Filho, s/n, Bloco G2-042, Ilha do Fundão, Rio de Janeiro 21941-902, Brazil; 3 Department of Surgical Sciences and Integrated Diagnostics (DISC), University of Genoa, 16132 Genova, Italy; 4 Anesthesia and Intensive Care, IRCCS Azienda Ospedaliera Metropolitana, 16132 Genova, Italy

**Keywords:** acute respiratory distress syndrome (ARDS), sedation, mechanical ventilation, patient self-inflicted lung injury (P-SILI), respiratory physiology

## Abstract

In acute respiratory distress syndrome (ARDS), spontaneous breathing is often encouraged to preserve lung function, yet excessive patient-generated inspiratory effort can paradoxically worsen injury, a phenomenon termed patient self-inflicted lung injury (P-SILI). In mechanically heterogeneous lungs, uncontrolled effort amplifies regional stress, strain, and mechanical power, driving alveolar overdistension, cyclic recruitment–derecruitment, *pendelluft*, and inflammation. Cardiovascular consequences include elevated right ventricular afterload, impaired left ventricular filling, and exacerbation of pulmonary edema. While moderate spontaneous breathing may improve aeration in mild injury, evidence shows that vigorous effort in severe ARDS accelerates histological damage and regional lung stress. Early bedside assessment of respiratory drive and inspiratory effort identifies patients at highest risk, enabling targeted interventions. Strategies to preserve protective spontaneous breathing while limiting injurious effort include individualized positive end-expiratory pressure, titrated sedation, prone positioning, and short-term neuromuscular blockade. By integrating continuous physiological monitoring with personalized ventilatory management, clinicians can mitigate P-SILI, protect the diaphragm, and optimize outcomes. Future studies are needed to test physiology-guided interventions and establish evidence-based approaches to safely harness the benefits of spontaneous breathing in ARDS.

## 1. Introduction

Acute respiratory distress syndrome (ARDS) remains one of the most challenging conditions in critical care, characterized by acute hypoxemic respiratory failure and substantial morbidity and mortality worldwide [1,2]. Large observational studies indicate that ARDS accounts for approximately 10% of all intensive care unit (ICU) admissions and nearly one quarter of patients requiring mechanical ventilation [3]. In contemporary practice, however, ARDS is increasingly recognized and treated before the initiation of invasive mechanical ventilation. The expanding use of noninvasive respiratory support has broadened the clinical spectrum of ARDS to include a substantial proportion of spontaneously breathing patients, a population that was underrepresented in earlier definitions and clinical paradigms [4,5]. In the LUNG SAFE study, approximately one third of patients with ARDS exhibited spontaneous breathing on the first day of ICU admission [3]. These data indicate that, in many patients, the early phase of ARDS unfolds under conditions of active respiratory drive, a factor likely to influence the subsequent trajectory of lung injury and respiratory failure.

This evolution in case mix has occurred in parallel with major changes in ventilatory management. Among patients who require invasive mechanical ventilation, current practice increasingly favors lighter sedation and a more restrictive use of neuromuscular blocking agents, allowing earlier preservation or reintroduction of spontaneous breathing during ventilatory support [6]. While these strategies aim to reduce complications associated with deep sedation and diaphragmatic inactivity, they also place renewed emphasis on the physiological consequences of patient effort in the injured lung.

Against this background, increasing attention has focused on the concept of patient self-inflicted lung injury (P-SILI), whereby excessive or poorly regulated inspiratory effort may aggravate pre-existing lung damage [7]. Although the extent to which P-SILI independently shapes the clinical course of ARDS remains debated, both experimental and clinical studies consistently indicate that respiratory drive and inspiratory effort are major determinants of lung stress and injury progression in acute hypoxemic respiratory failure [8]. This perspective extends the traditional focus on ventilator-induced lung injury (VILI) and highlights the need to consider the patient and the ventilator as a coupled system, in which injurious forces may originate from either or both components [9].

Recent advances in bedside physiological monitoring have further strengthened interest in identifying patients at risk of effort-related lung injury and in individualizing ventilatory support accordingly [10,11]. Nevertheless, important uncertainties remain regarding the clinical relevance of P-SILI, the reliability and interpretability of available monitoring tools, and the optimal strategies for balancing the potential benefits of spontaneous breathing against the risk of exacerbating lung injury [12]. Clinicians continue to face unresolved questions about when patient effort should be encouraged, when it should be limited, and how these decisions should be guided at the bedside.

In this review, we synthesize current experimental and clinical evidence on P-SILI in ARDS, examine available approaches for assessing respiratory drive and inspiratory effort, and discuss practical strategies for clinical management. We also highlight key gaps in knowledge and propose directions for future research aimed at refining physiology-guided, individualized respiratory support in this population.

## 2. Pathophysiological Mechanisms

**Figure 1:**
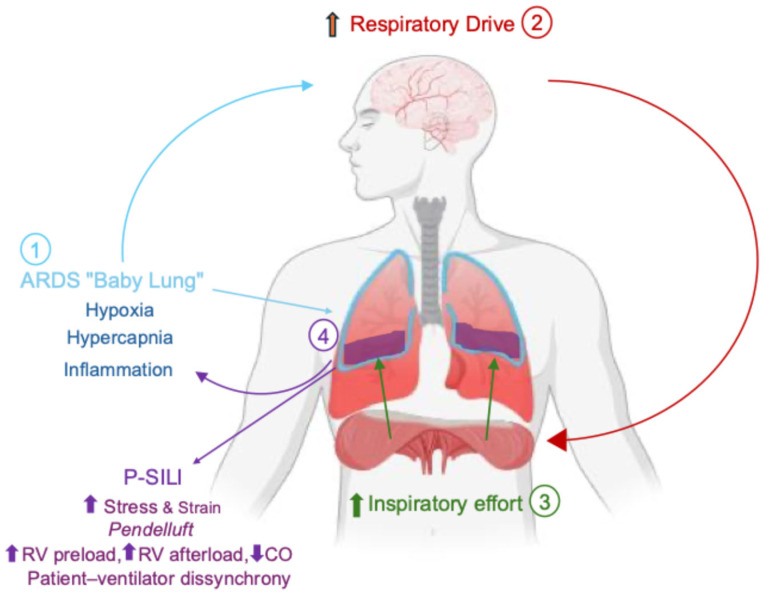
**Vicious Cycle Driving Patient Self-Inflicted Lung Injury (P-SILI).** Schematic representation of the self- pathophysiological cycle that promotes P-SILI in acute respiratory distress syndrome (ARDS). Lung injury reduces the functional aerated lung volume (“baby lung”), impairing gas exchange and leading to hypoxemia and hypercapnia, which increase respiratory drive and are further modulated by inflammatory mediators. Excessive respiratory drive generates vigorous inspiratory effort, which amplifies lung stress and strain, promotes pendelluft, patient–ventilator dyssynchrony, inflammation, and adverse hemodynamic effects, including increased right ventricular (RV) preload and afterload and reduced cardiac output (CO). These mechanisms further aggravate lung injury and gas exchange impairment, thereby sustaining and amplifying the vicious cycle of P-SILI.

Understanding P-SILI requires integrating the biomechanical and physiological consequences of excessive respiratory effort in the setting of a markedly reduced functional lung. In ARDS, patient-generated inspiratory effort acts on a structurally heterogeneous respiratory system, leading to disproportionate regional transmission of forces and amplification of local mechanical stress, thereby worsening lung injury [13,14]. These processes interact in a self-perpetuating cycle in which impaired gas exchange increases respiratory drive, further intensifying tissue stress and injury (Figure 1) [15].

### 2.1. Stress and Strain: The Mechanical Foundation of Effort-Related Injury

**Figure 2:**
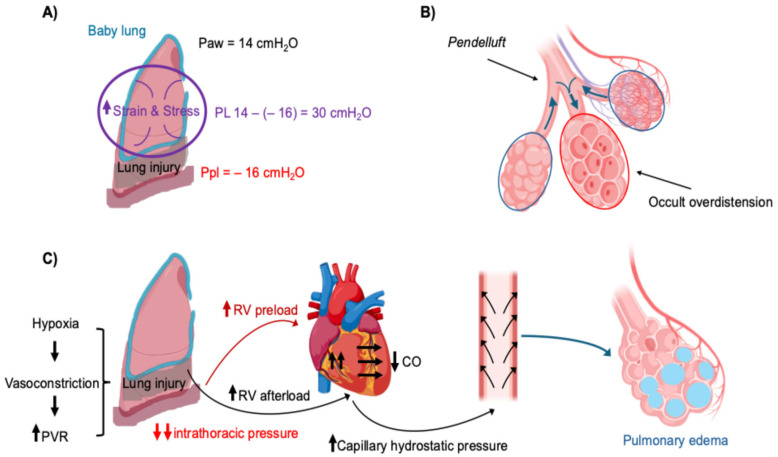
**Mechanisms of Effort-Related Lung and Cardiovascular Injury During Spontaneous Breathing.** (**A**) Mechanical effects of vigorous inspiratory effort on the “baby lung” in ARDS. During spontaneous breathing, patient-generated negative pleural pressure (Ppl) adds to airway pressure (Paw), resulting in a marked increase in transpulmonary pressure (PL) despite apparently protective ventilator settings. This amplification of lung stress and strain promotes alveolar overdistension and mechanical injury. (**B**) Pendelluft during spontaneous breathing in heterogeneous ARDS. Intrapulmonary gas shifts from non-dependent to dependent lung regions during early inspiration, leading to excessive regional tidal volume and occult overdistension without changes in global tidal volume. (**C**) Lung–heart interactions during intense inspiratory effort. Large negative intrathoracic pressure increases right ventricular (RV) preload, while hypoxia-induced pulmonary vasoconstriction increases pulmonary vascular resistance (PVR) and RV afterload. As RV systolic pressure rises, ventricular interdependence limits left ventricular filling, reducing cardiac output (CO) and systemic oxygen delivery. Elevated RV pressures also increase pulmonary capillary hydrostatic pressure, particularly in dependent lung regions exposed to high mechanical stress, thereby accelerating pulmonary edema formation.

A defining feature of ARDS is the marked reduction in aerated lung volume, giving rise to the so-called “baby lung” [16]. In spontaneously breathing patients, large negative intrathoracic pressure swings generated to maintain ventilation distribute a given tidal volume (VT) across a much smaller parenchymal volume, thereby amplifying lung stress—defined by transpulmonary pressure (PL), and strain, expressed as volume change relative to functional residual capacity [17]. Even when VT is set within the protective range (6–8 mL/kg predicted body weight [PBW]), vigorous inspiratory efforts can markedly increase PL beyond what is apparent from airway pressure measurements, imposing a concealed mechanical burden on the lung (Figure 2A) [10,17].

When neuromuscular function is preserved, increases in respiratory drive translate directly into greater inspiratory effort and higher PL, often with little change in arterial partial pressure of oxygen (PaO_2_) and carbon dioxide (PaCO_2_) [18]. The resulting PL reflects the combined effects of positive airway pressure and the negative pleural pressure generated by the patient. When excessive, this combined load may reach injurious levels and further damage the “baby lung” [15], establishing a vicious cycle in which escalating effort worsens lung injury, reduces compliance, and further increases respiratory drive.

Excessive strain promotes alveolar overdistension, disruption of the interstitial matrix, and failure of the epithelial–endothelial barrier, leading to pulmonary edema and worsening ventilation–perfusion mismatch [14]. In parallel, cyclic recruitment and derecruitment of unstable lung units generate high shear forces at air–tissue interfaces, particularly in dependent and structurally heterogeneous regions [19,20]. These biomechanical disturbances activate mechanotransduction pathways that amplify inflammation and increase vascular permeability [20,21,22,23]. Together, these mechanisms underscore the need for accurate assessment and timely limitation of excessive respiratory effort as a core component of lung-protective management.

### 2.2. Lung Heterogeneity and Regional Mechanical Load

ARDS is characterized by marked regional heterogeneity in aeration, compliance, and perfusion, which provides the physiological basis for the concept of superimposed pressure. Dependent lung regions commonly exhibit edema and alveolar collapse, whereas non-dependent areas remain relatively well aerated [19]. This configuration creates stress concentration at the interface between aerated and nonaerated tissue, predisposing these transitional zones to mechanical injury [24].

During spontaneous breathing, this heterogeneity is further amplified by uneven pleural pressure gradients, resulting in higher PL in dependent than in non-dependent regions [25].

One important manifestation of this process is *pendelluft*, characterized by intrapulmonary gas redistribution from non-dependent to dependent regions early in inspiration, driven by delayed expansion of stiff dorsal lung units in addition to the inspiratory flow delivered from the airway (Figure 2B). Although global VT may remain unchanged, regional tidal excursions can double or triple, producing occult overdistension that is not detected by conventional ventilator monitoring [26].

Consistent with this mechanism, an experimental study in lung-injured pigs has shown that dorsal VT during spontaneous breathing can be nearly threefold higher than during passive ventilation under neuromuscular blockade [26]. These findings suggest that substantial, and potentially injurious, regional PL can develop during spontaneous breathing, even when global VT and driving pressure (ΔP) appear to be within protective limits.

In addition, the large negative pleural pressure swings generated by intense inspiratory effort increase transmural pressure across the pulmonary microvasculature, thereby promoting fluid transudation into the interstitium and alveolar space. This effort-induced pulmonary edema further accentuates lung heterogeneity, impairs gas exchange, and reinforces the physiological drivers of excessive respiratory effort [27]. Lung heterogeneity thus becomes both a cause and a consequence of P-SILI.

### 2.3. Lung–Heart Interaction: Hemodynamic Consequences of Strong Inspiratory Effort

Cardiovascular interactions represent a central component of the pathophysiology of P-SILI. During spontaneous breathing, large negative swings in intrathoracic pressure increase venous return and right ventricular (RV) preload [28]. At the same time, pulmonary vascular resistance is frequently elevated in ARDS because of hypoxic vasoconstriction, vascular obstruction, and microthrombosis, thereby increasing RV afterload [29]. The combination of increased preload and afterload may precipitate acute RV dilation and dysfunction [29,30].

As RV pressures rise, ventricular interdependence limits left ventricular (LV) filling, reducing cardiac output and systemic oxygen delivery [31]. The resulting deterioration in PaO2 further stimulates respiratory drive, thereby sustaining a self-reinforcing cycle of vigorous inspiratory effort and hemodynamic compromise [8,31]. In parallel, elevated RV and pulmonary capillary pressures increase hydrostatic forces within the pulmonary microcirculation, particularly in dependent lung regions already exposed to high mechanical stress, thereby accelerating pulmonary edema formation (Figure 2C) [29,30,31].

Pulmonary vascular integrity may be further compromised by cyclic increases in transmural vascular pressure during intense inspiratory effort, promoting capillary stress failure and facilitating the extravasation of red blood cells into the alveolar space. This process not only contributes to edema formation but also amplifies local inflammation and oxidative injury. Through these tightly coupled mechanisms, cardiovascular strain emerges as an active contributor to lung injury during forceful spontaneous breathing, rather than merely a downstream consequence of respiratory failure [27].

### 2.4. Patient–Ventilator Interaction: Synchrony and Intensity of Mechanical Ventilation

In partially assisted ventilation, the instantaneous coupling between patient effort and ventilator assistance determines the effective mechanical load imposed on the lung [32]. From a physiological perspective, the sum of ventilator-applied pressure and patient-generated pleural pressure swings defines the dynamic PL profile, thereby shaping tidal stress, strain, and the mechanical power delivered to the respiratory system [9].

When respiratory drive becomes excessive, due to hypoxemia, metabolic acidosis, agitation, or impaired neuromechanical coupling, patient–ventilator interaction often deteriorates, and the combined effort–assist pattern may rapidly become injurious [33,34].

In this context, dyssynchrony represents a key pathway through which excessive drive is translated into harmful mechanical loading. It is clinically and physiologically relevant because it amplifies pressure swings, increases dynamic strain, and promotes unstable breath-to-breath variability. Together, these effects increase both the intensity and the frequency components of mechanical power, thereby favoring the development of P-SILI [8,33,34,35].

Although fully controlled ventilation reliably abolishes excessive spontaneous effort, it usually requires deep sedation or neuromuscular blockade, which are associated with well-recognized complications, including ventilator-induced diaphragm dysfunction (VIDD), impaired secretion clearance, delirium, and delayed weaning [36,37,38]. Partial neuromuscular blockade has therefore been proposed as a physiological compromise, attenuating excessive inspiratory effort while preserving some degree of respiratory muscle activation. From a mechanistic standpoint, modulation of muscle activity represents a means to control lung stress when respiratory drive cannot be adequately reduced by ventilatory adjustments or sedation alone [6]. The clinical objective, therefore, is not to suppress spontaneous breathing per se, but to achieve a lung- and diaphragm-protective balance: preserving physiologically appropriate effort while preventing excessive drive, minimizing dyssynchrony, and limiting injurious mechanical forces.

## 3. Experimental Evidence

Preclinical models have been central to establishing the mechanistic basis of P-SILI. Sustained spontaneous hyperventilation has been shown to induce severe lung injury even in previously healthy lungs, characterized by extensive, poorly recruitable atelectasis, whereas no comparable damage occurs under controlled mechanical ventilation [39]. Complementary experiments in isolated lung preparations have demonstrated that hyperventilation alone is sufficient to trigger inflammatory mediator release, underscoring the intrinsic injurious potential of excessive respiratory drive [40].

Subsequent studies in experimental lung injury have clarified that the impact of spontaneous breathing critically depends on both the intensity of inspiratory effort and the severity of the underlying lung damage. During assisted ventilation, high inspiratory effort increases PL and preferentially aggravates structural injury in dependent lung regions. In models of mild lung injury, spontaneous breathing can improve oxygenation and aeration by redistributing VT toward dependent areas [41]. In contrast, in severe lung injury, spontaneous breathing is associated with higher PL swings, greater cyclic end-expiratory collapse in dependent regions, no improvement in gas exchange, and more pronounced histological damage [42].

A distinct mechanism of effort-related injury, termed “occult *pendelluft*,” has been identified in injured lungs. In this setting, negative pleural pressures generated by diaphragmatic contraction redistribute gas from non-dependent to dependent regions, producing regional overstretch without any increase in global VT [26]. This reflects the solid-like mechanical behavior of injured lungs, with heterogeneous transmission of pleural pressure and non-uniform distending forces. As a result, inspiratory effort is disproportionately concentrated in dependent regions, where regional PL and stress are locally amplified [10].

Additional experimental work has shown that vigorous spontaneous breathing, compared with muscle paralysis, preferentially damages dependent lung regions. At low levels of positive end-expiratory pressure (PEEP), a steep vertical pleural pressure gradient promotes tidal recruitment and derecruitment of dependent lung units, generating regional strain comparable to that produced by very large VTs during controlled ventilation. Increasing PEEP reduces inspiratory effort, partly through electromechanical uncoupling of the diaphragm, attenuates the pleural pressure gradient, and decreases regional strain and inflammation [43]. Imaging studies further demonstrate that intrapulmonary gas redistribution can occur even before inspiratory flow begins, increasing regional stress without changes in global VT; higher PEEP markedly reduces these phenomena and supports its protective role against effort-related injury [44,45].

The interaction between spontaneous effort and inflammation is further highlighted in models combining resistive breathing with endotoxin-induced lung injury. While resistive breathing alone causes only modest physiological changes, its combination with inflammatory lung injury markedly worsens gas exchange, increases histological damage, and amplifies inflammatory signaling, supporting a mechanistic link between excessive mechanical stress and biotrauma [46].

Importantly, not all forms of spontaneous breathing are deleterious. In severe ARDS supported by extracorporeal assistance to control respiratory drive, a pattern characterized by low PL swings, high respiratory rate, elevated PEEP, and very low VTs increases dorsal ventilation without evidence of *pendelluft* or aggravated lung injury [47]. These observations emphasize that the injuriousness of spontaneous breathing is not inherent but depends on the magnitude and distribution of the mechanical forces generated.

Finally, growing evidence indicates that intense, unsupported breathing also imposes substantial mechanical stress on the diaphragm itself, accelerating muscle injury. Noninvasive continuous positive airway pressure has been shown to attenuate both lung and diaphragmatic damage in experimental models, supporting the concept of an integrated lung–diaphragm protective strategy [48,49]. More recent work suggests that tissue injury is governed not only by the magnitude of deformation (strain) but also by the rate at which it is applied (strain rate). By reducing both inspiratory and expiratory strain rates and improving diaphragmatic relaxation, positive airway pressure further limits mechanical stress on the respiratory muscles, reinforcing its physiological and protective rationale [49,50].

## 4. Clinical Experience

Clinical observations in patients with ARDS provide a coherent translational counterpart to experimental findings and are consistent with the clinical relevance of mechanisms encompassed by the concept of P-SILI. Although largely indirect, evidence from observational, physiological, and interventional studies consistently links excessive respiratory drive and inspiratory effort to disease progression and worse outcomes. Importantly, these associations persist after adjustment for conventional indices of severity, suggesting that uncontrolled spontaneous breathing is not merely a marker of illness severity but may contribute to the evolution of lung injury.

In the ACURASYS trial, early neuromuscular blockade in patients with moderate-to-severe ARDS improved adjusted survival and increased ventilator-free days without increasing muscle weakness [51]. Although inspiratory effort was not directly quantified, a plausible mechanistic explanation is the suppression of excessive spontaneous breathing and patient–ventilator dyssynchrony, phenomena now recognized as important contributors to regional lung stress, *pendelluft,* and occult overdistension [52]. Subsequent analyses suggested that this benefit is heterogeneous, being more pronounced in patients with more severe hypoxemia or more favorable baseline prognosis, and less evident in those with advanced age or a rapidly fatal trajectory [53]. In contrast, the ROSE trial, which used lighter sedation in the control group and higher levels of PEEP, did not demonstrate a survival benefit of routine early neuromuscular blockade and reported more adverse events [54]. These methodological differences are clinically relevant, as both higher PEEP and lighter sedation are associated with partial attenuation of respiratory drive and inspiratory effort, potentially reducing the contrast between treatment strategies [55].

More recently, a physiological proof-of-concept study showed that partial neuromuscular blockade during assisted ventilation can substantially reduce VT, PL, and diaphragmatic electrical activity while preserving some degree of spontaneous breathing. By selectively moderating inspiratory effort rather than abolishing it, this approach decouples respiratory drive from excessive lung stress and may facilitate lung-protective ventilation during partial ventilatory support [6]. Although preliminary and requiring outcome validation, these findings support the feasibility of targeted modulation of inspiratory effort as a strategy to balance lung and diaphragm protection.

In patients with moderate-to-severe ARDS, the maintenance of spontaneous breathing during non-invasive respiratory support appears particularly hazardous. Large observational cohorts have consistently shown that, in individuals with PaO_2_/FiO_2_ ratios below 150 mmHg, initial treatment with non-invasive ventilation (NIV) is frequently followed by failure, delayed intubation, and increased mortality. Propensity-adjusted analyses further indicate higher ICU mortality among patients managed initially with non-invasive strategies compared with those receiving early invasive mechanical ventilation [56]. These findings do not establish causality but are consistent with the hypothesis that, in severe lung injury, uncontrolled spontaneous breathing may amplify regional lung stress and strain, particularly in dependent lung regions, thereby aggravating structural damage [41,43].

Physiological studies in acute hypoxemic respiratory failure, a population that largely overlaps with ARDS, provide mechanistic insight into these clinical observations. Surrogate markers of elevated respiratory drive, such as high VT during NIV, are independently associated with treatment failure and the need for intubation, even after adjustment for hypoxemia and global severity of illness [57]. Similarly, the inability to achieve an early reduction in inspiratory effort, assessed by esophageal pressure swings shortly after initiation of non-invasive support, identifies patients at particularly high risk of deterioration [58].

Alterations in PaCO_2_ further reinforce the clinical relevance of excessive respiratory drive. Marked hypocapnia before or shortly after the initiation of NIV is associated with a substantially increased risk of intubation, and this relationship persists during the first hours of support [59]. In this context, hypocapnia likely reflects a maladaptive imbalance between ventilatory demand and the mechanical capacity of the injured lung, rather than an effective compensatory response, and may serve as a readily available clinical signal of potentially injurious respiratory effort.

More recent clinical investigations have attempted to estimate the mechanical stress imposed on the lung during spontaneous or assisted breathing by integrating airway and pleural pressure measurements. In patients with ARDS receiving NIV or CPAP, higher estimates of total lung stress are associated with failure of non-invasive support and the need for intubation. Longitudinal assessment reveals divergent trajectories: patients successfully managed without intubation show a progressive reduction in lung stress, whereas those who fail non-invasive strategies exhibit persistently elevated or increasing values [60]. These dynamic patterns mirror experimental observations in which sustained inspiratory effort perpetuates lung injury, whereas attenuation of effort mitigates regional stress and inflammation [42,43,44,45,46].

Large prospective observational data further support the clinical relevance of patient-generated lung stress during assisted ventilation. In a multicenter cohort of patients with acute hypoxemic respiratory failure managed with partial ventilatory support, higher driving pressures and lower respiratory system compliance during the first days of support were independently associated with ICU mortality, even though VT and direct indices of inspiratory effort did not differ between survivors and non-survivors. Over time, driving pressure progressively diverged between these groups, underscoring the dynamic relationship between mechanically relevant lung stress and outcome [61]. Although causality cannot be inferred, these findings suggest that elevated driving pressure during assisted breathing identifies patients at higher risk and may represent a meaningful target for monitoring and physiological modulation.

Taken together, clinical evidence closely parallels experimental observations. In established ARDS, particularly when lung injury is severe, sustained spontaneous breathing accompanied by elevated inspiratory effort is consistently associated with higher lung stress, greater regional injury, and worse outcomes. Conversely, strategies that effectively limit respiratory drive and inspiratory effort appear capable of mitigating mechanical injury. These data support the view that spontaneous breathing in ARDS is neither intrinsically protective nor intrinsically harmful, but strongly context-dependent, and underscore the need for careful monitoring and modulation of respiratory effort to minimize the risk of P-SILI.

## 5. Bedside Monitoring

Prevention of P-SILI depends on the timely recognition of excessive spontaneous breathing at the bedside. Because P-SILI arises from the interaction between neural respiratory drive, the resulting inspiratory muscle effort, and the mechanical properties of the injured lung, monitoring strategies should distinguish between these interdependent components rather than rely solely on global ventilatory variables. In parallel, early identification of potentially hazardous breathing patterns, including elevated VTs, marked patient–ventilator dyssynchrony, and spatially inhomogeneous ventilation, is essential to detect situations in which spontaneous breathing may become injurious.

### 5.1. Assessment of Respiratory Drive

Diaphragm electrical activity (EAdi), measured via a dedicated nasogastric catheter, reflects activation of the crural diaphragm and can be quantified using the peak amplitude, the inspiratory rise slope, and the neural inspiratory time. Because of substantial interindividual variability, EAdi is primarily useful for tracking trends over time rather than defining absolute thresholds of excessive drive [62,63]. Ratios between airway pressure or VT and EAdi provide indices of neuromuscular and neuroventilatory efficiency, allowing estimation of inspiratory muscle pressure and its pressure–time product [64,65,66]. A limitation of this approach is that EAdi does not capture the contribution of accessory respiratory muscles, which may become prominent as respiratory effort increases.

In intubated patients, airway occlusion pressure measured at 100 ms (P0.1) provides a robust bedside index of respiratory drive. P0.1 reflects the pressure generated by all inspiratory muscles during the first 100 ms of an inspiratory effort against an occluded airway and is largely independent of respiratory mechanics, muscle fatigue, and airway resistance [67,68]. Normal values typically range between 0.5 and 1.5 cmH_2_O, whereas values above approximately 3.5 cmH_2_O indicate excessive neural drive. Elevated P0.1 has been associated with dyspnea, prolonged mechanical ventilation, and increased mortality [69]. Large observational data further demonstrate a U-shaped relationship between P0.1 and outcome, with both abnormally low and abnormally high values associated with delayed ICU discharge, underscoring the importance of maintaining respiratory drive within a physiological range [70,71].

### 5.2. Assessment of Respiratory Effort

Esophageal pressure monitoring remains the reference method for assessing inspiratory effort [72]. During spontaneous breathing, contraction of the respiratory muscles generates a negative swing in esophageal pressure (ΔPes), which reflects changes in pleural pressure and thus the magnitude of inspiratory effort. Physiological ΔPes typically ranges between 5 and 10 cmH_2_O, whereas larger excursions indicate excessive effort [73,74]. From esophageal pressure measurements, inspiratory muscle pressure (Pmus) can be estimated by accounting for the elastic recoil of the chest wall, thereby isolating the active muscular contribution [8].

In patients with acute hypoxemic respiratory failure receiving different modes of ventilatory support, increasing Pmus is associated in a dose-dependent manner with higher transpulmonary driving pressure, larger VTs, and lower inspiratory alveolar pressure relative to PEEP, changes that define a mechanically unfavorable breathing pattern. Clinically, higher lung stress predicts subsequent deterioration in respiratory system compliance, whereas lower inspiratory alveolar pressure is associated with impaired oxygenation [75].

More integrative indices of inspiratory effort include the work of breathing (WOB), defined as the integral of Pmus over volume, and the pressure–time product (PTP), defined as the integral of Pmus over time. Both metrics quantify the energetic burden imposed on the respiratory muscles and provide a more comprehensive assessment of effort than isolated pressure or volume measurements [76].

In intubated patients, the airway pressure deflection measured during an end-expiratory occlusion (ΔPocc) provides a practical surrogate of inspiratory effort and correlates with both inspiratory muscle pressure (Pmus) and esophageal pressure swing (ΔPes) [77,78]. In patients with respiratory failure, Pmus and ΔPes have been estimated at approximately 0.75 × ΔPocc and 0.66 × ΔPocc, respectively. Both abnormally low and abnormally high ΔPocc values, reflecting insufficient or excessive respiratory effort, have been associated with higher ICU mortality and a lower probability of ICU discharge, particularly in patients with more severe hypoxemia [71].

During spontaneous breathing, cyclic changes in intrathoracic pressure generate corresponding oscillations in central venous pressure. When esophageal pressure monitoring is unavailable, tidal variations in central venous pressure (ΔCVP) can therefore be used as a surrogate of ΔPes and provide an indirect estimate of inspiratory effort [79]. In a preliminary clinical study, a ΔCVP threshold of 8 mmHg predicted a ΔPes greater than 10 cmH_2_O. In patients with a pulmonary artery catheter, respiratory variations in pulmonary artery occlusion pressure may reflect intrathoracic pressure changes more accurately than ΔCVP, further improving assessment of inspiratory effort [80,81].

An emerging non-invasive approach is measurement of the nasal pressure swing (ΔPnose), which reflects the maximal negative pressure generated during spontaneous inspiration and transmitted to the upper airway. ΔPnose can be obtained using a nasal cannula connected to a pressure transducer. Preliminary data show a close correlation with ΔPes and suggest that elevated values are associated with failure of non-invasive respiratory support [82,83,84].

An additional metric is the Pressure–Muscle Index (PMI), defined as the difference between plateau airway pressure (Pplat) and peak airway pressure delivered by the ventilator. PMI reflects the contribution of the patient’s inspiratory muscles relative to ventilator support and respiratory system compliance (Crs) [8]. A PMI < 0 cmH_2_O—when Pplat is lower than peak airway pressure—indicates minimal inspiratory effort (PTP/min < 50 cmH_2_O·s/min; Pmus < 5 cmH_2_O) and suggests ventilatory over-assistance. Although thresholds for excessive effort are less well defined, PMI is particularly useful for identifying patients at risk of over-assistance and impaired respiratory drive [85,86,87].

Diaphragm ultrasonography, particularly the diaphragm thickening fraction (TFdi), provides a non-invasive estimate of diaphragmatic activation [88,89]. Although its quantitative relationship with ΔPes varies with loading conditions and diaphragm integrity, very low values suggest over-assistance or suppressed effort, whereas high values indicate excessive loading and potential risk of load-induced diaphragmatic injury [90,91]. While TFdi does not measure inspiratory effort directly, it remains a practical tool for tracking changes in respiratory muscle activity and guiding ventilator adjustment [8]. For clinical applicability, Table 1 summarizes commonly used bedside thresholds for respiratory drive, inspiratory effort, and lung stress that may help identify patients at increased risk of P-SILI.

## 6. Risk Situations for P-SILI and Clinical Management

From a pathophysiological perspective, P-SILI results from a maladaptive interaction between respiratory drive, inspiratory effort, lung mechanical impairment, and ventilatory assistance [13,14,15]. Although spontaneous breathing is highly prevalent in ARDS, particularly in the early phases of the disease, its impact is strongly context dependent. While preserved effort may be beneficial in mild lung injury, converging experimental and clinical evidence shows that, as compliance decreases and hypoxemia worsens, vigorous inspiratory effort generates injurious PL swings, regional overdistension, and increased mechanical power, thereby accelerating lung injury [26,41,42,43,44,45,46,47,51,54,56,57,58,59,60,61].

Non-invasive respiratory support represents a paradigmatic risk scenario. Although CPAP and NIV can improve oxygenation and recruit lung units, neither strategy reliably suppresses respiratory drive in moderate-to-severe ARDS [57,58,59,92]. When inspiratory effort remains high, the combination of patient-generated negative pleural pressure and externally applied airway pressure produces large PL swings and elevated mechanical power. A similar phenomenon occurs during assisted mechanical ventilation, where excessive patient effort may summate with ventilator assistance and generate injurious patterns despite apparently protective airway pressures and VTs [10,17,93]. Patient–ventilator dyssynchrony further amplifies breath-to-breath variability and regional stress. Across these settings, persistently elevated inspiratory effort identifies patients at increased risk of P-SILI and should prompt immediate reassessment of ventilatory strategy [8,33,34,35].

Clinical management therefore requires a physiology-based approach aimed not at abolishing spontaneous breathing per se, but at shaping inspiratory effort to match respiratory demand to the mechanical capacity of the injured lung. In mild disease, spontaneous breathing can usually be preserved with appropriate monitoring. In moderate-to-severe ARDS, however, prevention of effort-related lung injury becomes a clinical priority [94].

Optimization of ventilatory settings, particularly PEEP, plays a central role. By improving lung homogeneity, increasing end-expiratory lung volume, and reducing cyclic recruitment, adequate PEEP lowers inspiratory threshold load and attenuates pleural pressure swings, thereby reducing regional strain and dynamic lung-distending pressure [55]. These mechanisms explain why higher PEEP can mitigate effort-related injury even in the presence of intrapulmonary gas redistribution, and why total dynamic lung-distending pressure, rather than *pendelluft per se*, appears to be the dominant determinant of injury risk [43,44,55,95,96].

**Figure 3:**
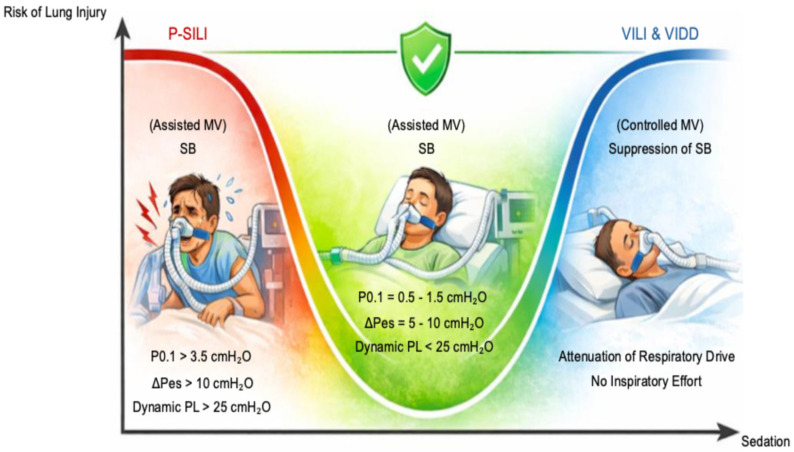
**Effect of Sedation on the Risk of Lung Injury during Mechanical Ventilation.** Schematic representation of the U-shaped relationship between sedation depth and the risk of lung injury. Inadequate sedation permits excessive respiratory drive, reflected by elevated P0.1 (>3.5 cmH_2_O), large inspiratory effort (ΔPes > 10 cmH_2_O), and increased mechanical stress (dynamic transpulmonary pressure, PL > 25 cmH_2_O), thereby promoting patient self-inflicted lung injury (P-SILI). At the opposite extreme, deep sedation suppresses spontaneous breathing, rendering the patient passive and increasing the risk of ventilator-induced lung injury (VILI) and ventilator-induced diaphragm dysfunction (VIDD) during controlled ventilation. An intermediate, carefully titrated level of sedation attenuates respiratory drive and inspiratory effort (P0.1 = 0.5–1.5 cmH_2_O; ΔPes = 5–10 cmH_2_O) and limits mechanical stress (dynamic PL < 25 cmH_2_O), while preserving spontaneous breathing and positioning the patient within the protective zone of this U-shaped relationship. This figure was created with ChatGPT-5.2.

Sedation is a key tool to modulate excessive respiratory drive and improve patient–ventilator interaction, but its effects follow a U-shaped relationship. Insufficient sedation permits excessive drive and injurious effort, whereas deep sedation abolishes effort entirely, exposing the patient to VILI and VIDD [94]. Intermediate titration can reduce respiratory drive, inspiratory effort, and dynamic PL without eliminating spontaneous breathing, thereby occupying the protective middle of this physiological continuum [97] (Figure 3).

Therefore, lung protection should be complemented by diaphragm protection. Both excessive inspiratory effort and abolition of spontaneous breathing are harmful, promoting load-induced diaphragmatic injury and VIDD, respectively. A diaphragm-protective approach targets a physiological range of inspiratory effort, aligning respiratory muscle preservation with lung-protective ventilation [36,37,38,48,49,50].

In patients with severe ARDS and persistently high drive despite optimization of ventilatory settings and sedation, short courses of neuromuscular blockade may be required to interrupt the cycle of effort-induced lung injury. Duration should be minimized to limit diaphragm disuse and ICU-acquired weakness [98].

Prone positioning provides an additional protective mechanism. By improving lung homogeneity, reducing dorsal collapse, and redistributing ventilation toward better-perfused regions, pronation reduces stress concentration and regional strain and often improves oxygenation, which may secondarily reduce respiratory drive. Although proning alone cannot fully neutralize the effects of vigorous spontaneous breathing, it represents a powerful adjunct within a comprehensive lung-protective strategy [99,100,101].

Overall, prevention of P-SILI hinges on continuous assessment of inspiratory effort and dynamic lung stress, recognizing that both excessive effort and injurious passivity can be harmful. Clinical strategies should therefore aim to dynamically balance respiratory drive, ventilatory assistance, and lung mechanics to preserve protective breathing while avoiding self-inflicted lung injury.

## 7. Knowledge Gap and Future Directions

Despite growing recognition of P-SILI, several fundamental uncertainties remain. ARDS is increasingly understood as a heterogeneous syndrome encompassing distinct biological and clinical subphenotypes [102,103]. In this context, uncontrolled spontaneous breathing likely represents a relevant, but largely unmeasured, source of additional heterogeneity. In clinical practice, no patient receives immediately optimized ventilatory support at the onset of respiratory failure, leaving a variable early window during which patient-generated inspiratory effort interacts with a structurally and mechanically heterogeneous lung. Under these conditions, experimental and clinical data indicate that regional differences in compliance and pleural pressure transmission produce highly uneven distributions of stress and strain, potentially accelerating injury in vulnerable lung units [26,41,42,43,44,45,58].

Although several bedside tools are now available to characterize respiratory drive, inspiratory effort, and regional ventilation during spontaneous or assisted breathing, their use remains largely confined to physiological studies. Esophageal pressure monitoring allows estimation of PL and provides direct insight into the mechanical load imposed on the lung by the interaction between patient effort and ventilatory assistance [104]. In parallel, techniques such as electrical impedance tomography permit continuous, non-invasive assessment of regional ventilation distribution, enabling detection of ventilation heterogeneity, *pendelluft*, and regional overdistension [105].

The combined application of these approaches offers a unique opportunity to move beyond global airway pressures and VTs and to directly observe, in real time, how respiratory drive, inspiratory effort, and lung mechanics interact within the injured lung. However, these tools have not yet been integrated into routine clinical decision-making, and there is currently no high-quality evidence demonstrating that physiology-guided strategies improve patient-centered outcomes. A critical next step is therefore the design of interventional trials that test ventilatory strategies guided by PL, inspiratory effort, and regional ventilation patterns, rather than by airway pressure and VT alone. Such an approach may enable more precise, individualized respiratory support that adapts to both patient-specific physiology and the dynamic evolution of lung injury.

## 8. Conclusions

Patient self-inflicted lung injury represents a clinically important and potentially modifiable contributor to the progression of ARDS. Available experimental and clinical evidence indicates that spontaneous breathing is neither inherently protective nor inherently harmful; its effects depend on the severity of lung injury, the magnitude of respiratory drive, and the interaction between patient effort and ventilatory support.

Prevention of P-SILI therefore requires continuous attention to respiratory drive, inspiratory effort, and the resulting dynamic stress applied to the lung. Clinical management should aim to preserve spontaneous breathing when it remains within safe physiological limits, while promptly limiting excessive effort through a combination of optimized PEEP, carefully titrated sedation, prone positioning, and, when necessary, short-term neuromuscular blockade.

A physiology-based, individualized approach, centered on matching respiratory effort and ventilatory assistance to the mechanical capacity of the injured lung, provides the most coherent framework to protect both lung and diaphragm while minimizing the risk of self-inflicted injury.

## Figures and Tables

**Table 1:** Clinically relevant bedside thresholds to assess respiratory drive, inspiratory effort, and lung stress in patients at risk of P-SILI.

Variable	Physiological/Target Range	Range Suggestive of Excessive Effort or Stress	Clinical Interpretation
**P0.1** **(cmH** **_2_** **O)**	0.5–1.5	>3.5	Indicates elevated neural respiratory drive, associated with dyspnea, prolonged ventilation, and worse outcomes
**ΔPes** **(cmH** **_2_** **O)**	5–10	>10	Reflects excessive inspiratory effort and increased pleural pressure swings, associated with higher lung stress
**ΔPocc (cmH** **_2_** **O)**	3.5–7	>7	Surrogate of excessive inspiratory effort when esophageal pressure is unavailable
**ΔCVP** **(mmHg)**	0–7	>8	Surrogate of excessive inspiratory effort when esophageal pressure is unavailable
**ΔPnose** **(cmH** **_2_** **O)**	5–10	>10	Surrogate of excessive inspiratory effort when esophageal pressure is unavailable
**Dynamic PL (cmH** **_2_** **O)**	<25	>25	Suggests excessive lung stress and increased risk of lung injury, even with protective airway pressures

## Data Availability

No new data were created for the writing of this manuscript.

## Abbreviations

The following abbreviations are used in this manuscript:

ACURASYS	*ARDS et Curarisation Systématique* (trial)
ARDS	Acute Respiratory Distress Syndrome
CPAP	Continuous Positive Airway Pressure
CVP	Central Venous Pressure
ΔCVP	Tidal variation of Central Venous Pressure
ΔPes	Esophageal pressure swing
ΔPocc	Airway pressure deflection during end-expiratory occlusion
ΔP	Driving pressure
EAdi	Electrical Activity of the Diaphragm
FiO_2_	Fraction of Inspired Oxygen
ICU	Intensive Care Unit
LV	Left Ventricle
NIV	Non-Invasive Ventilation
PaCO_2_	Arterial partial pressure of carbon dioxide
PaO_2_	Arterial partial pressure of oxygen
PBW	Predicted Body Weight
PEEP	Positive End-Expiratory Pressure
PL	Transpulmonary Pressure
Pmus	Inspiratory Muscle Pressure
P-SILI	Patient Self-Inflicted Lung Injury
P0.1	Airway occlusion pressure at 100 ms
PTP	Pressure–Time Product
ROSE	*Reevaluation Of Systemic Early neuromuscular blockade* (trial)
RV	Right Ventricle
TFdi	Diaphragm Thickening Fraction
VIDD	Ventilator-Induced Diaphragm Dysfunction
VILI	Ventilator-Induced Lung Injury
VT	Tidal Volume
WOB	Work Of Breathing
